# Spare the Birds

**Published:** 1878-11

**Authors:** 


### SPARE THE BIRDS.

Many a fond association hovers around the familiar name of Robin Redbreast; he is the loved harbinger of the coming Spring-time. In early March, while the snow still lingers in the fields, on stake, or fence, or bending twig, he perches himself, and utters his well-loved notes. Brigand with the dog and gun! touch not a feather; harm him not a bit. By all the memories of departed childhood, be his protector and his friend!

It seems a savage law, at first sight, that in England, a man should be sent to prison for killing a bird; but it is as much an act of vandalism, as to pluck a beautiful flower in a garden not one's own. The sweet notes of a single bird, in the course of a season, may soothe a hundred hearts; in another hundred, may wake up strong yearnings for the innocence of childhood; and in a hundred more, may arouse to cheerful activities hearts almost crushed by bitter disappointments and by wasting cares; while not one of any hundred will fail of a pleasant feeling at the first sound of its voice. Is not he a vandal, then, who kills a robin, and without any single compensation for the act, shuts off a thousand pleasurable emotions from the hearts of his toiling brother man? But, let us appeal from sentiment to cents.

Forty years ago, it was customary to have a shooting-match on May election day; and on one of these, so many birds were killed, that a farmer purchased them by the cart-load to manure his fields; and the scarcity of birds, that season, was a subject of general remark. A few weeks later, the grass withered, and blackened, and died.

If the gizzard of a robin is examined, daily, for two months of early Spring, there will not be found a single particle of vegetable food; but it will be found full of a fleshy material. In every gizzard will be found from one to two hundred insects; these, multiplied by sixty days for every robin, and that product again by the vast multitudes of that sweet bird, which swarm in innumerable thousands from New Hampshire to the Carolinas, the actual destruction of insect life becomes amazing; and when it is calculated that each insect, in common with its class, if permitted to live, is the parent of thousands more for another season, the actual amount of riddance performed by the robin alone, for any one year, is more than an army of men could accomplish with the aid of millions of money.

Men who have worked in the surface earth much, have sometimes, with one stroke of the spade or hoe, loosened myriads of whitish, sluggish, winged insects. These insects, while under ground, feed on the roots of grasses and berries, withdrawing from them all their substance, leaving the plant to die.

It is upon these under-ground insects and larvæ that the robin feeds in early Spring, when they begin to wake up to life. His instincts and his activities give him their joint aid in ferreting out these hordes of destroyers, and he feeds upon them heartily.

Who shall deny that the first love of blood is planted in the bosom of a truant school-boy, in his first forage against the beautiful and innocent warblers of the wood, to ripen, as manhood comes on, into an unfeeling temper, and a savage, murderous conduct? No doubt, the bootless and remorseless destruction of a bird has ended in the pitiless murder of a man. Fishing and hunting are no educators of the affections; but they do feed some of the worst traits of our nature, and cannot but tend to callous the heart.

